# Annotations

**Published:** 1900-12-22

**Authors:** 


### ANNOTATIONS.

### Habitual Inebriates.

A CASE came before the magistrate at the Westminster police court last week which illustrates forcibly the evil results of the delay which has taken place in providing accommodation for inebriates under the provisions of the Act of 1898. It was the case of a woman who had been convicted 33 times of drunkenness. The magistrate said that under the Inebriates Act he had the power to send a person like this away for three years, but that practically nothing had yet been done in London to give effect to the provisions of the Act. Things, he said, were at an absolute deadlock, there being no homes available. The case before him was a typical one, a woman for whom everything had been tried. She had been sent away over and over again for a month with hard labour, she had been bound over in sureties for six months, remaining in gaol for that period, and she had even been sent to the sessions, and dealt with there as a rogue and vagabond. All, however, to no purpose. "She leaves prison one day and is here again the next," said the magistrate, and he added "If I could avail myself of the powers which the legislature in its wisdom decreed, I could send her away for three years—long enough to reform body and soul." Surely something might be done to hurry up those who are responsible for making provision for the proper carrying out of the provisions of this Act, an Act which stands almost alone in providing means for reclaiming the habitual drunkard. It was passed in the year 1898, and the matter of reformatories has been under the consideration of the London County Council for more than a year. It is now many months since the Committee to which the question had been referred reported to the County Council that having regard to the insufficiency of the accommodation for Protestant women at the Duxhurst Reformatory, and the difficulties they had met with in other directions, the Fairfield Estate had been secured, and arrangements were being pushed forward as quickly as possible for the adaptation of the buildings on the estate for the purposes of a reformatory for females. The hope was then expressed that by June of this year the reformatory would be ready; and it is distinctly disappointing to find a magistrate stating now in December that matters are still at such an absolute deadlock that he cannot find a place to which he can send the inebriates who come before him.

### Wanted a Sanitary Standard.

IT is earnestly to be hoped that so soon as the new municipalities to which the local government of London is now so largely entrusted have settled down to the work before them they will seriously consider the sanitary standard which they are prepared to enforce, and will endeavour to introduce greater uniformity of practice in such matters than has hitherto prevailed. The variety of views taken by the different vestries as to the extent to which their officials were to be encouraged, or even to be allowed, to put the sanitary law in operation against offenders has sometimes amounted to a positive scandal. To a certain extent, of course, the sanitary authorities are at the mercy of the magistrates in regard to the suppression of nuisances, but no one can be blind to the differences of practice which prevail in different parts, and we cannot but feel that if the newly-appointed bodies would but co-operate in laying down a standard to which they would all conform, and would but agree to put into practice to the full all those powers which are placed in their hands by the sanitary law a great change would soon come over the health, and perhaps the morals, of the population. In going across London one has long been accustomed to see differences in the treatment of the streets as one passed from one district to another—different kinds of lighting, different methods of scavenging, different standards of cleanliness. This has been open to the eyes of everyone. In the course of a short walk one could pass through three or four independent districts, each ruled by an independent vestry, each of which, with entire indifference to surrounding areas, managed its own affairs in its own independent way. What we would point out is that this independence has gone far deeper than mere matters of street lamps and street sweeping; that, in fact, it has extended through the whole system of sanitary administration, and that not merely the methods adopted, but the standards aimed at by the forty-three authorities which have hitherto been the rulers of London have had but small relation to each other. Now, however, there would seem to be a great opportunity for introducing a reform in the direction of common aims even if not of common methods in sanitation, and it is to be hoped that it will not be neglected.

### An Imperial Pharmacopœia.

AT the present moment the drift of affairs is all towards Imperialism, and thus it is but appropriate that even in the preparation of a supplement to the Pharmacopœia this tendency has not been overlooked. We have received an advanced copy of the Indian and Colonial Addendum to the British Pharmacopœia which, we may fairly take it, is but a step towards a consummation which would be highly fitting to the dawn of a new century, namely, the inclusion within one single Pharmacopœia of all the formulæ for the preparation of medicines throughout the whole area of the Queen's dominions. At present the confusion is great, and its extent may be judged by the statement that in its endeavour to adapt this new Addendum to the requirements of the Empire at large, the Medical Council received expressions of concurrence from each of the seventy Administrations of Her Majesty's dominions. It is no light task to co-ordinate the requirements of seventy separate administrations in different parts of the globe, practically representing seventy countries, each with its own group of native remedies for the treatment of its own indigenous diseases. Nevertheless, no one can doubt that it is only proper and right that one weight, one measure, and one recipe for the preparation of the drugs used in medicine should hold good throughout the Empire, and we welcome this earnest of an Imperial pharmacopœia all the more heartily because its preparation would seem to suggest the possibility that some time or another we may hope to see even an international pharmacopœia rise in its place. This may, perhaps, be a dream, but few things would tend more to break down the barriers between practitioners of different countries than the establishment of uniformity in the medicines they employ, and if an Empire on which the sun never sets, and within the bounds of which every latitude and every climate is represented, can once show the practicability of an Imperial pharmacopœia, a pharmacopœia for the whole world ought not to be an impossibility.

